# Surgical induction model of femoral defect in Wistar rats for bone repair histology

**DOI:** 10.1590/1414-431X2025e15168

**Published:** 2026-03-30

**Authors:** F.L.D. de Oliveira, A.C. Nagato, T.N. França, F.M. Aarestrup, B.J.V. Aarestrup

**Affiliations:** 1 Programa de Pós-Graduação em Medicina Veterinária (Patologia e Ciências Clínicas), Universidade Federal Rural do Rio de Janeiro, Seropédica, RJ, Brasil; 2 Laboratório de Biofísica, Morfofisiologia e Inflamação, Instituto de Ciências Biológicas, Universidade Federal de Juiz de Fora, Juiz de Fora, MG, Brasil; 3 Departamento de Biofísica e Fisiologia, Instituto de Ciências Biológicas, Universidade Federal de Juiz de Fora, Juiz de Fora, MG, Brasil; 4 Departamento de Epidemiologia e Saúde Pública, Instituto de Veterinária, Universidade Federal Rural do Rio de Janeiro, Seropédica, RJ, Brasil; 5 Faculdade de Ciências Médicas e da Saúde / Suprema – Serviço de Alergia e Imunologia da Maternidade Therezinha de Jesus, Juiz de Fora, MG, Brasil; 6 Departamento de Morfologia, Instituto de Ciências Biológicas, Universidade Federal de Juiz de Fora, Juiz de Fora, MG, Brasil

**Keywords:** Research design, Surgery, Femur, Bone defect, Histology

## Abstract

The creation of bone lesions using a well-established surgical technique is essential for obtaining histological images in experimental models that aim to study the pathophysiology of bone repair. In this study, we report the steps of a surgical method to induce bone defects in Wistar rats, providing a basis for experimental studies requiring precision in these lesions, especially for future experimental tests and subsequent histological analysis. The animal model remains an important alternative for investigating the inflammatory process and bone regeneration. The model consists of creating experimental, non-critical surgical bone defects in the femurs of Wistar rats (*Rattus norvegicus*, 90 days old, weighing between 250-300 g) using a 2-mm spherical drill and a low-speed motor. The present report includes reproducible information on the selection of the animals and the materials needed to perform anesthesia and surgery to induce bone defects. It also provides details on post-surgical care, sample collection, and preparation of samples for histological processing. The steps presented here can significantly increase the accuracy of lesion creation and allow for more precise results in the analysis of bone architecture and repair.

## Introduction

Bone defects are often associated with fractures, osteoporosis, osteotomies, bone neoplasms, osteomyelitis, and consolidation complications (1). They can also be induced experimentally to investigate the course of repair (regeneration without an increase in the volume of newly formed bone) or bone regeneration (mineralization process with an increase in bone volume), which are events not yet fully understood (2). In experimental models, efforts have been made to standardize and classify the methods used to create bone defects (3); however, the methods used vary greatly (4), which can significantly affect the results of experimental outcomes.

Rodents are commonly used in preclinical research involving bone repair for several reasons: a) they are easy to reproduce in the laboratory in a short period of time; b) there is a wide availability of reagents for histochemistry (3), molecular biology, immunohistochemistry, and oxidative stress and damage (5,6) assays; c) they are less costly in terms of care and management compared to medium and large animals (7,8); d) both homogeneous and heterogeneous strains are available (9-11); e) they share anatomical and physiological characteristics, biomechanics, and inflammatory (12) and immune responses with humans (13-15); and f) most importantly, they have a faster metabolism and healing time (16) and tolerate surgery well (1,16-18).

Specifically, bones of Wistar rats (*Rattus norvegicus*) are used as a model for testing biomaterials, implants, and for studies investigating therapeutic effects capable of accelerating the repair process (19-23). Bone defects in the cortical bone of the femoral diaphysis in Wistar rats, in particular, are commonly used in studies involving greater mechanical load because these bones are more resistant compared to skulls (16,17). Femurs are easier to access surgically and, from a mechanical point of view, are free from compensatory load distribution between adjacent bones, as occurs between the radius and ulna or the tibia and fibula (16,24). Furthermore, bone defects in the femoral diaphysis exhibit fewer complications because they do not affect the epiphyses, which would result in greater mechanical instability and increased complexity of the anatomical structures involved (3).

The creation of bone defects requires technical skill, as refined methods minimize interference from poorly executed surgical techniques (4), improve the reproducibility of experimental models, increase the possibility of comparison between studies, and help avoid unnecessary repetition of experiments (25). Good practice in these cases goes beyond the choice of animal model (26) and involves determining the geometry of the bone defect to be induced (3).

In general, the edges of bone lesions are characterized by complex inflammatory microenvironments, and the lack of dimensional control of these lesions can add an additional variable to the study. Drills attached to low-speed motors are used to create circular bone defects of known diameter (22,26-28). Approximating the lesion edges favors bone scaffold formation and inflammatory cell migration, which accelerate repair (29), while lesions with larger diameters tend to have a more prolonged repair process (30).

A wide variety of experimental models have been used to investigate the course of bone repair. However, in bone lesions with variable geometry, there is no clarity regarding the details of the experimental and surgical procedures that enable the reproducibility of the methods used (15,21-23,31-39), especially concerning the pre-, peri-, and post-surgical stages of femoral bone defect induction using low-speed drill motors. The model proposed here details the pre-, peri-, and post-surgical phases that enable surgical reproducibility by systematizing the use of clear, well-defined methods for inducing standardized bone defects (without fracture and excessive bleeding and with adequate exposure of the medullary canal), which is lacking in the literature. Notably, the rigorous use of these methods ensures that researchers obtain a well-defined bone defect geometry in an anatomically resistant region with less compensatory load distribution between adjacent bones (i.e., with mechanical stability) in an diaphyseal anatomical bone region without involvement of epiphyseal structures (i.e., with fewer complications), which improves the control of variables in the experimental model. Thus, the objective of this report was to present the methods used to create surgical bone defects in the femurs of Wistar rats using a drill and a low-speed motor for histological evaluation of bone repair.

## Material and Methods

### Ethics statement and animal maintenance

The use of animals for the creation of bone defects was evaluated and approved in accordance with the Ethical Standards of the National Guidelines on the Care and Use of Laboratory Animals from the Brazilian National Council for the Control of Animal Experimentation (CONCEA) and the principles of the International Council for Laboratory Animal Science (ICLAS). All experimental methods were approved by the Ethics Committee on Animal Use of the Federal University of Juiz de Fora and registered under number 033/2016.

**Figure 1:**
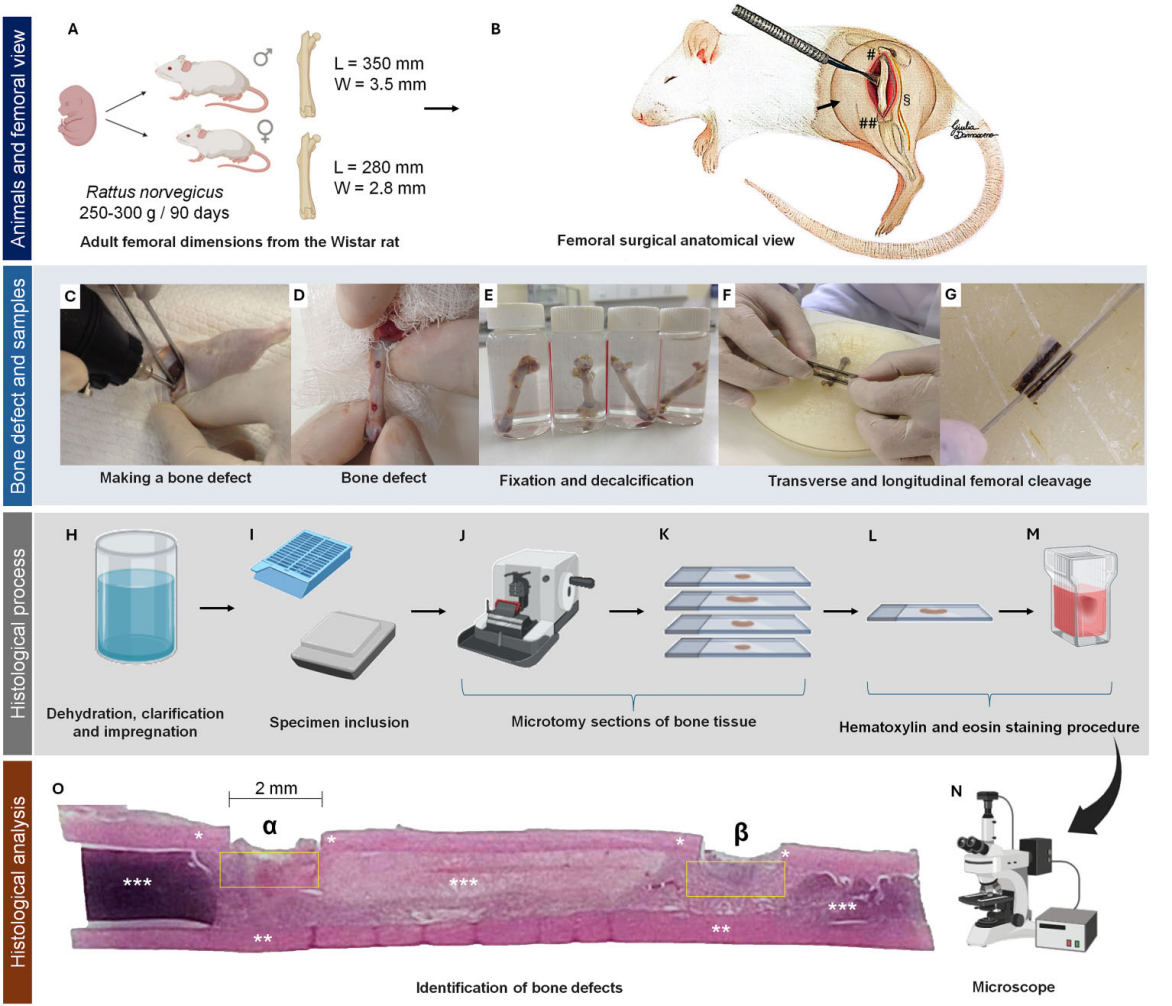
Summary of the methods used for the surgical creation of bone defects using drills. **A**, Female and male femoral dimensions of the adult Wistar rat (250-300 g/90 days). L: Length; W: Width. **B**, Anatomical view of the hip (#) and knee (##) joints and the sciatic nerve (§) parallel to the longitudinal axis of the femur. **C**, Creation of the bone defect with the 2-mm drill bit attached to the motor over the bone diaphysis. **D**, Two equidistant bone defects in the femoral diaphysis. **E**, Fixation (10% calcium formalin for at least 24 h) and decalcification (5% nitric acid solution for 72 h) of specimens. **F**, Transverse and (**G**) longitudinal femoral cleavage process. **H**-**M**, Histological process. **N**, Light microscope coupled with an image capture system. **O**, Section of bone tissue showing the proximal (α) and distal (β) bone defects, cis (*) and trans (**) cortical bone, (***) bone marrow, and bone neoformation area (yellow rectangle).

After weaning at 21 days of age, the pups were randomized (the number of animals depends on each experimental design) and individually placed into mini-isolators measuring 501 (L) × 264 (H) × 341 mm (W), lined with wood shavings, in ventilated racks, at a controlled temperature of 22±2°C, under a 12-h light/dark cycle, with sterile pellet feed and filtered water *ad libitum*, until they reached 90 days of age, weight between 250 and 300 g, and approximately 23 cm (males) or 16 cm (females) in length (from nose to anus). At this age, the adult femoral length in males is approximately 350 mm (measured from the upper boundary plane - greater trochanter - to the lower boundary plane - lateral condyle) and 3.5 mm wide (at the central diaphyseal cranial face). In adult females, the femur is approximately 280 mm long and 2.8 mm wide, respectively (Figure 1A).

### Establishment of the animal model

**Figure 2:**
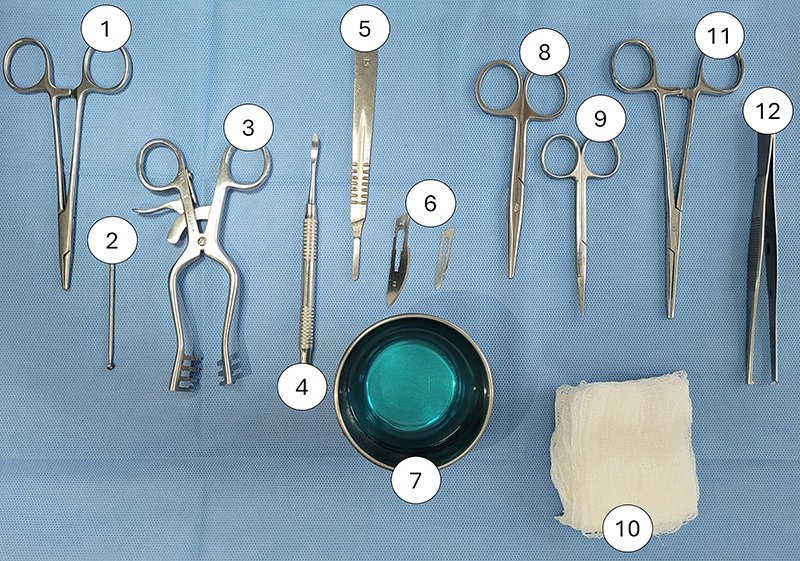
Materials required for performing the surgical approach and creating the bone defect in the femoral diaphysis of Wistar rats: 1) Mayo-Hegar needle holder; 2) spherical carbide surgical drill (2 mm); 3) Weitlaner self-tapping retractor; 4) Freer periosteum elevator; 5) scalpel handle; 6) scalpel blades; 7) vat containing 0.5% alcoholic chlorhexidine; 8) Metzembaum baby scissors; 9) Iris scissors; 10) gauze; 11) Kelly hemostatic forceps; 12) rat tooth dissecting forceps.

Prior to the surgical procedure, the following materials were listed, procured, and checked. This started with materials for the surgeon's personal use: gloves, a cap, a mask, and a gown. Next were the materials for preparing and monitoring the anesthesia and analgesia stages for the animals: a scale, 0.5% single-use chlorhexidine, cotton, a 1-mL syringe, 10% ketamine hydrochloride, 2% xylazine hydrochloride, a heating pad, fentanyl citrate, and buprenorphine hydrochloride. Materials for the surgical procedure included a silent trichotomy machine, a 1-L bottle of 0.5% single-use chlorhexidine, 16 pairs of sterile surgical gloves, 16 caps, 16 masks, 16 sterile surgical gowns, 16 sterile surgical drapes, 4 scalpel handles (No. 3), 16 scalpel blades (No. 15), 4 Metzenbaum scissors, 4 anatomical dissecting forceps, 4 Freer periosteal elevators, 4 small self-retaining retractors, 4 sterile surgical rulers (in mm), 4 tungsten carbide spherical surgical drills (2 mm diameter), 1 low-speed micromotor, 176 1-mL syringes, 1 500-mL bottle of 0.9% sodium chloride solution, 100 sterile gauze compresses, 16 absorbable 400 surgical sutures, 16 non-absorbable 400 sutures, and 1 surgical glue (Figure 2). These materials were estimated for 16 surgical procedures on 16 animals, totaling 32 bone defects, and should be adjusted for each experimental design.

**Figure 3:**
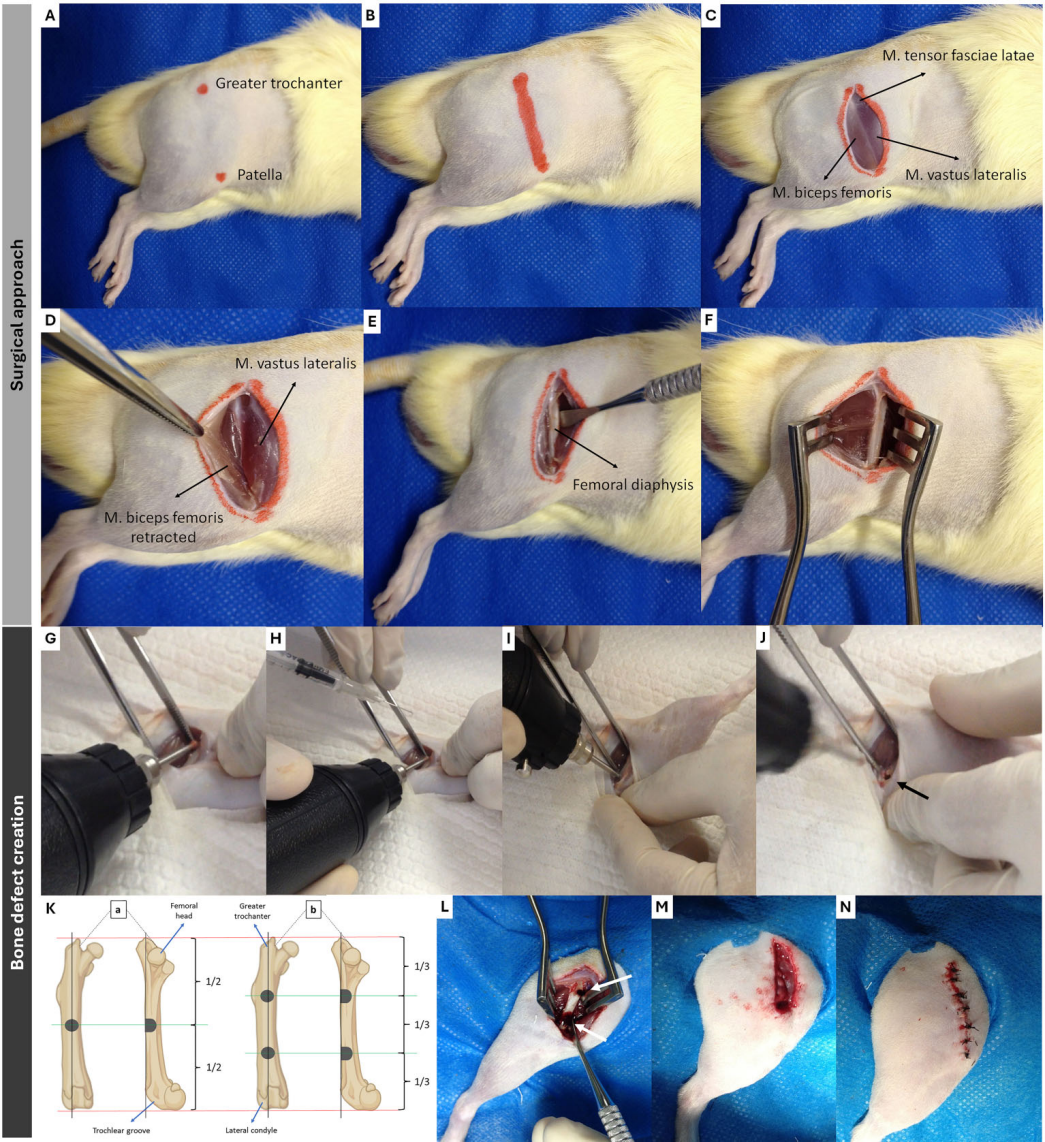
Surgical approach and creation of bone defects in the right femoral diaphysis of a male Wistar rat (*Rattus norvegicus*) aged 90 days and weighing 250-300 g. **A**, Trichotomy (4-cm margin) and identification of femoral anatomical landmarks - red dots indicate the greater trochanter (proximally) and the patella (distally). **B**, Site of skin incision (red line). **C**, Tensor fasciae latae muscle (proximally), vastus lateralis (cranially), and biceps femoris (caudally) are exposed. **D**, Caudal retraction (rat-tooth forceps) of the biceps femoris muscle. **E**, Exposure of the femoral diaphysis (with the Freer periosteal elevator). **F**, Maintenance of femoral diaphysis view (with Weitlaner self-retaining retractor). **G**, Initial (oblique) positioning of the motor-burr unit relative to the long axis of the femur. **H**, Surgical irrigation with 0.9% NaCl (with a 1-mL syringe and 27×0.7 mm needle). **I**) Repositioning of the motor-burr unit (perpendicular) in relation to the long axis of the femur. **J**, Single bone defect created in the femoral diaphysis (black arrow). **K**, Illustrations of craniocaudal and mediolateral femoral views for planning the number of bone defects: a, creation of a single defect; b, creation of two equidistant defects. **L**, View of the bone defects (white arrows). **M**, *Vastus lateralis* muscle suture. **N**, Skin suture. **A**-**J**, *Ex vivo* animal model. **L**-**N**, *In vivo* animal model.

Anesthesia began with the preparation of the anesthetic solution, calculated based on the animal's weight. This involved adding a volume corresponding to 90 mg/kg of 10% ketamine hydrochloride and 10 mg/kg of 2% xylazine hydrochloride to a single 1-mL syringe. After the animal was adequately restrained manually, the abdomen was antiseptically prepared with 2% chlorhexidine digluconate, and the anesthetic solution was administered intraperitoneally (*ip*) into the right lower quadrant of the abdomen. Ten to 15 min were allowed for the animal to become anesthetized, with the depth of anesthesia confirmed by relaxation of jaw muscle tone, absence of the palpebral reflex, and absence of response to tail stimulation or interdigital reflexes (40). Once these parameters were confirmed, the animal was placed on the heating pad in lateral recumbency, with the temperature set between 35.8 and 37.7°C. Body temperature was monitored using an intrarectal thermometer. Next, extensive trichotomy was performed on the entire limb and its peripheral region with a radius of approximately 4 cm (Figure 3A and B), followed by removal of excess hair and antisepsis with 0.5% alcoholic chlorhexidine. A sterile surgical drape with a circular opening was placed over the area to isolate the limb, especially the surgical region, from the inguinal and perineal areas.

The surgical procedure began with a longitudinal incision along the lateral surface of the femur using a No.15 scalpel blade. The greater trochanter was used as the anatomical reference for the latero-proximal aspect, and the patella was used as the reference for the cranio-distal aspect (Figure 3A and B). After dissecting the subcutaneous tissue, the vastus lateralis, biceps femoris, and tensor fasciae latae muscles were exposed through this incision (Figure 3C). The fascia lata was then incised to reflect the vastus lateralis cranially and the biceps femoris caudally (Figure 3D), exposing the femoral diaphysis (Figure 3E). The exposure was maintained by positioning a self-retaining retractor (Figure 3F).

Note 1: Adequate prior anatomical knowledge is important in order to preserve the sciatic nerve, which is located caudal to the biceps femoris muscle (Figure 1B). Damage to this nerve may result in neuropraxia or neurotmesis, which can significantly compromise postoperative recovery.

A single intraperitoneal (*ip*) injection of fentanyl citrate (0.03 mg/kg) was administered before creating the bone defect. Then, slight external rotation of the femur was performed to expose the cranial surface of the diaphysis, where the bone defect was created. First, the drill was positioned at an oblique angle (Figure 3G) under constant irrigation with 0.9% saline solution (Figure 3H). Then, the drill was positioned perpendicular to the femoral diaphysis (Figure 3I) until the defect was complete (Figure 3J). To standardize the location of the defect, a straight line was drawn along the long axis of the femur, starting from the center of the greater trochanter and extending toward the lateral portion of the trochlear groove. The distance between the ends was divided into two or three portions to determine the intersection point of the center of the defect with the initial line (Figure 3K). After creating the defect (Figure 3L), the musculature and subcutaneous tissue were approximated using a simple continuous suture with absorbable thread (Figure 3M). The skin was then closed with a simple interrupted suture using non-absorbable thread and surgical glue was applied (Figure 3N).

Note 2: A 0.9% saline solution was used for irrigation throughout the time the drill was in contact with the bone to prevent heat-induced osteolysis at the edge of the defect.

Note 3: In the present model, the bone defect is considered complete when the drill breaks through the first bone cortex (cis) and exposes the medullary canal, thus generating a monocortical defect model.

Note 4: Each surgery takes a trained professional approximately 45 to 60 min, which helps to estimate the time required for the experiments. The surgeon's technical skill and prior knowledge of anatomy can reduce surgical time and avoid iatrogenic injury.

During the immediate postoperative period, each animal was transferred to an individual cage with controlled temperature and received an injection of the analgesic buprenorphine hydrochloride (0.1 mg/kg). The animals were then monitored until they could voluntarily return to sternal recumbency, after which they were able to move around. After this stage, water and food intake was monitored over the following hours and days. The analgesic was administered every 12 h for four consecutive days, and the surgical wound was cleaned daily with 0.9% saline solution.

At the end of the experimental period (Figure 1C), the animals were euthanized in accordance with ethical guidelines. After dissection of the surrounding tissue, the femur was collected, followed by disarticulation of the coxofemoral (hip) and femoro-tibio-patellar (knee) joints (Figure 1D). For histological processing, the femurs were fixed in a 10% calcium formalin solution for at least 24 h (Figure 1E) and decalcified in a 5% nitric acid solution for at least 72 h (Figure 1E). The samples were then subjected to transverse microtomy of the epiphyses (Figure 1F) and longitudinal microtomy of the diaphysis (Figure 1G). The specimens underwent gradual alcohol dehydration, xylene clarification, and paraffin embedding (Figure 1H). Next, 4- and 5-µm slices were cut using a microtome (Figure 1I-K) and deposited on appropriate glass slides (Figure 1L). The slides were then stained with hematoxylin and eosin (HE) (Figure 1M).

**Figure 4:**
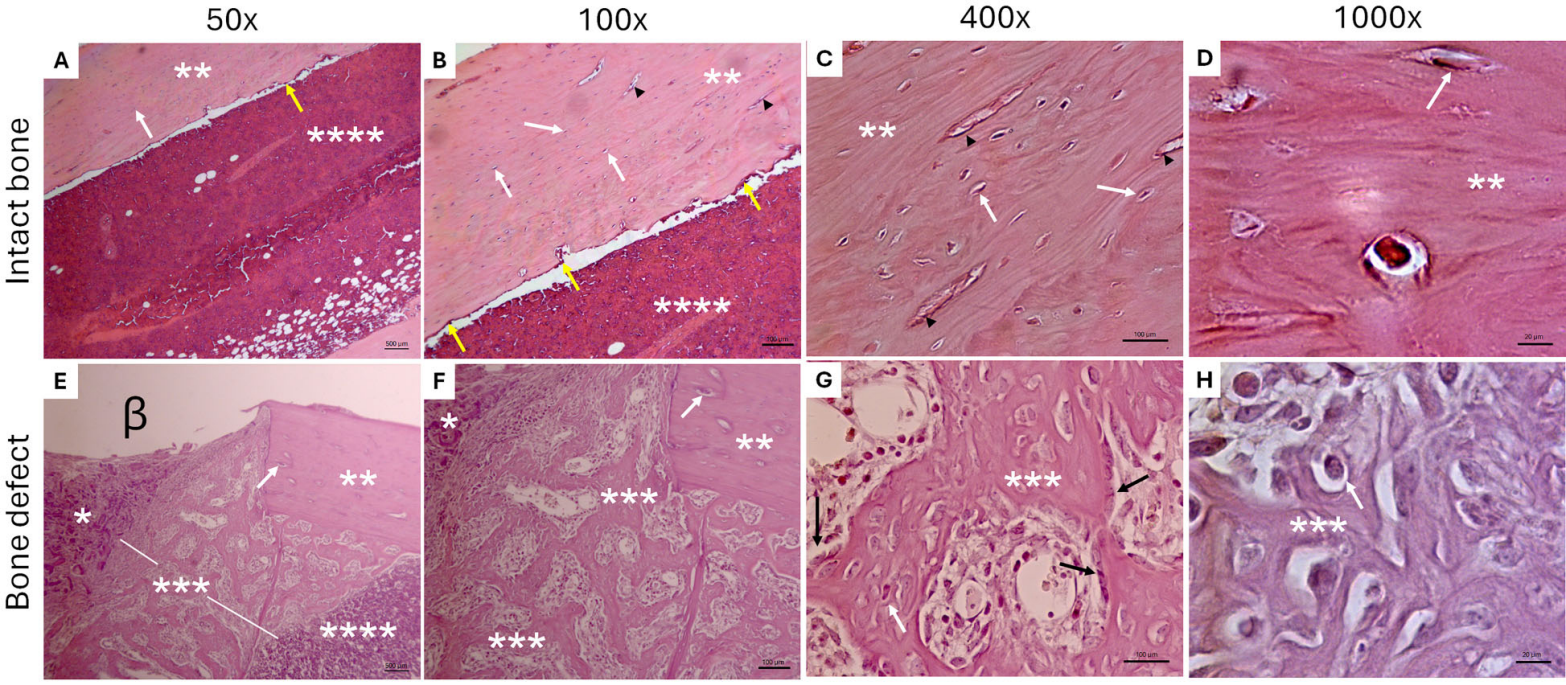
Photomicrographs of bone tissue of 90-day-old male Wistar rats (*Rattus norvegicus*) weighing between 250 and 300 g stained with hematoxylin and eosin. **A**-**D**, Mature and defect-free femoral cortical bone of a control animal. **A** and **B**, Preserved medullary canal (**A**=50×; **B**=100×). **C** and **D**, Mature bone cortex (**C**=400×; **D**=1000×). **E**-**H**, Bone defect in the process of repair with newly formed bone (**E**=50×; **F**=100×; **G**=400×; **H**=1000×). Scale bars=500, 100, 100, 20 μm. β: Bone defect; *inflammatory infiltrate; **bone cortex; ***area of newly formed bone; ****bone marrow; black arrowhead: blood vessels. Black arrows indicate osteoblasts, yellow arrows indicate lining cells, and white arrows indicate osteocytes.

The morphology of bone tissue repair areas was analyzed using a light microscope (Carl Zeiss Microscopy GmbH, 2011; Germany) equipped with an image capture system (Zeiss Axiocam CHF5) connected to a computer (Figure 1N). Proprietary software (Zeiss ZEN Lite image capture program for Windows - 2.3 Blue Edition) was used to activate real-time visualization mode and capture the microscopic fields of interest (Figure 1O α e β). Contrast was adjusted to ensure that bone cells could be clearly differentiated from inflammatory cells (see Figure 4). Basophilic nuclei were distinguished from the acidophilic bone matrix in at least five to ten randomly selected microscopic fields for probabilistic modelling. To this end, the slides were scanned at magnifications of 50, 100, 400, and 1000× to enable semi-automated histomorphometry and stereology of the bone neoformation areas (Figure 4).

Note 5: For the microscopic analysis of the sections, a Zeiss AxioCam CHF5 microscope (Germany) was used. The AxioCam camera, coupled to the Zeiss microscope, fully digitized the histological sections, as did the ZEN Lite image capture program for Windows (2.3 Blue Edition) - both of which were located in the Immunopathology and Experimental Pathology Laboratory of the CBR at UFJF.

Note 6: Histological differences, including cell population variety and morphological and morphometric alterations, can be analyzed using morphometry and stereology techniques.

## Discussion

In this study, we detailed the steps to produce standardized 2-mm diameter femoral diaphyseal bone defects using a drill, with adequate exposure of the medullary canal in Wistar rats - an animal model whose anatomical dimensions are well-suited for experimental surgeries. In addition, a wide variety of reagents are available for use with this type of rodent in experimental research. This expands the possibilities for studies that use bone lesions as a base model for further investigations into inflammation, oxidative stress, immune responses, and testing of prostheses, resins, and implants (3,11,14,18,19,20,22,24,34,36).

A diaphyseal femoral bone defect is recommended because it is anatomically resistant, provides greater mechanical stability with less compensatory load distribution between adjacent bones, and does not involve epiphyseal structures, which reduces complications. Creating bone defects with a drill minimizes intervening variables present in the complex microenvironment of lesions induced by less controllable bone injury methods. The use of Gigli saws (15), for example, does not guarantee cutting precision, and oscillating saws are costly (29,35). Saws generate segmental or wedge-shaped bone defects, requiring implants to stabilize bone fragments, whereas drill-induced bone defects create a tunnel or circumferential window in the bone, eliminating the need for stabilizing plates that can impair neovascularization at the bone regeneration site (3), thus increasing the risk of excessive bleeding. In addition, drills are versatile, accessible, and available in a wide range of diameters.

From a histological point of view, macroscopically induced bone defects require methodological rigor to create controlled tissue-level lesions with histoarchitecture that allows histomorphometric and stereological quantification. This approach allows determination of whether there is a loss of histoarchitectural integrity, as seen in fractures and diseases that modify bone matrix density, such as osteoporosis, rheumatoid arthritis, and neoplasms. Our group has previously demonstrated differences in density of newly formed in femoral diaphysis lesions created using drills (22). We highlight the importance of having a well-established technique so that experimental models investigating bone remodeling can benefit from these methods.

Methodologically induced bone defects, as shown here, allow for the reproducibility of macroscopic bone defects and directly reduce variability in experimental variables at the microscopic level, such as the density of osteocytes, osteoblasts, osteoclasts, and Haversian canals - parameters that must be considered morphometrically to help determine the state of bone maturation. Taken together, these reflections highlight the ongoing need to reevaluate and refine the research methods, including those used to create experimental bone defects.

## Data Availability

All data generated or analyzed during this study are included in this published article.

## References

[1] Horner EA, Kirkham J, Wood D, Curran S, Smith M, Thomson B (2010). Long bone defect models for tissue engineering applications:
criteria for choice. Tissue Eng Part B Rev.

[2] Hu K, Olsen BR (2016). The roles of vascular endothelial growth factor in bone repair
and regeneration. Bone.

[3] Sun Y, Helmholz H, Willumeit-Römer R (2022). Surgical classification for preclinical rat femoral bone defect
model: standardization based on systematic review, anatomical analysis and
virtual surgery. Bioengineering (Basel).

[4] Islam MA, Kamarrudin NS, Daud R, Noor SNFM, Azmi AI, Razlan ZM (2022). A review of surgical bone drilling and drill bit heat generation
for implantation. Metals.

[5] Castro MML, Nascimento PC, Souza-Monteiro D, Santos SM, Arouck MB, Garcia VB (2020). Blood oxidative stress modulates alveolar bone loss in
chronically stressed rats. Int J Mol Sci.

[6] Camilli AC, de Godoi MA, Costa VB, Fernandes NAR, Cirelli G, da Silva LKF (2024). Local application of a new chalconic derivative (Chalcone T4)
reduces inflammation and oxidative stress in a periodontitis model in
rats. Antioxidants (Basel).

[7] Nunamaker DM (1998). Experimental models of fracture repair. Clin Orthop Relat Res.

[8] Sparks DS, Saifzadeh S, Savi FM, Dlaska CE, Berner A, Henkel J (2020). A preclinical large-animal model for the assessment of
critical-size load-bearing bone defect reconstruction. Nat Protoc.

[9] Manassero M, Decambron A, Huu Thong BT, Viateau V, Bensidhoum M, Petite H (2016). Establishment of a segmental femoral critical-size defect model
in mice stabilized by plate osteosynthesis. J Vis Exp.

[B10] Breschi A, Gingeras TR, Guigó R (2017). Comparative transcriptomics in human and mouse. Nat Rev Genet.

[11] Huang EE, Zhang N, Shen H, Li X, Maruyama M, Utsunomiya T (2022). Novel techniques and future perspective for investigating
critical-size bone defects. Bioengineering (Basel).

[12] Claes L, Recknagel S, Ignatius A (2012). Fracture healing under healthy and inflammatory
conditions. Nat Rev Rheumatol.

[13] Kanczler JM, Ginty PJ, White L, Clarke NMP, Howdle SM, Shakesheff KM (2010). The effect of the delivery of vascular endothelial growth factor
and bone morphogenic protein-2 to osteoprogenitor cell populations on bone
formation. Biomaterials.

[B14] Srouji S, Ben-David D, Kohler T, Muller R, Zussman E, Livne E (2011). A model for tissue engineering applications: femoral critical
size defect in immunodeficient mice. Tissue Eng Part C Methods.

[15] Ueno M, Lo CW, Barati D, Conrad B, Lin T, Kohno Y (2020). Interleukin-4 overexpressing mesenchymal stem cells within
gelatin-based microribbon hydrogels enhance bone healing in a murine long
bone critical-size defect model. J Biomed Mater Res A.

[16] Gao H, Huang J, Wei Q, He C (2023). Advances in animal models for studying bone fracture
healing. Bioengineering.

[B17] Bigham-Sadegh A, Oryan A (2015). Selection of animal models for pre-clinical strategies in
evaluating the fracture healing, bone graft substitutes and bone tissue
regeneration and engineering. Connect Tissue Res.

[18] Wancket LM (2015). Animal Models for evaluation of bone implants and devices:
comparative bone structure and common model uses. Vet Pathol.

[19] Harada N, Watanabe Y, Sato K, Abe S, Yamanaka K, Sakai Y (2014). Bone regeneration in a massive rat femur defect through
endochondral ossification achieved with chondrogenically differentiated MSCs
in a degradable scaffold. Biomaterials.

[B20] Sato K, Watanabe Y, Harada N, Abe S, Matsushita T, Yamanaka K (2014). Establishment of reproducible, critical-sized, femoral segmental
bone defects in rats. Tissue Eng Part C Methods.

[B21] Brassolatti P, de Andrade ALM, Bossini PS, Orth DL, Duarte FO, Dos Anjos Souza AB (2018). Photobiomodulation on critical bone defects of rat calvaria: a
systematic review. Lasers Med Sci.

[B22] de Oliveira FLD, Nagato AC, Aarestrup FM, Aarestrup BJV (2022). Bone neoformation induced by low-level laser and methylene blue
suggests early ossification in rats. J Lasers Med Sci.

[23] Berni M, Brancato AM, Torriani C, Bina V, Annunziata S, Cornella E (2022). The role of low-level laser therapy in bone healing: systematic
review. Int J Mol Sci.

[24] McGovern JA, Griffin M, Hutmacher DW (2018). Animal models for bone tissue engineering and modelling
disease. Dis Model Mech.

[25] Lee KH, Lee DW, Kang BC (2020). The ‘R’ principles in laboratory animal
experiments. Lab Animal Res.

[26] Pritchett-Corning KR, Luo Y, Mulder GB, White WJ (2011). Principles of rodent surgery for the new surgeon. J Vis Exp.

[B27] Subelza PH, Kopp G, Sivila MFH, Sivila HKH, Francischone CE (2019). Comparative study of conventional drill bits and a new model for
low-rotation in the surgical bed preparation in bone blocks for installation
of dental implants. J Young Pharm.

[28] Bromer FD, Brent MB, Thomsen JS, Bruel A (2022). Drill-hole bone defects in animal models of bone healing:
protocol for a systematic review. JMIR Res Protoc.

[29] Alidadi S, Oryan A, Bigham-Sadegh A, Moshiri A (2017). Role of platelet gel embedded within gelatin scaffold on healing
of experimentally induced critical-sized radial bone defects in
rats. Int Orthop.

[30] Nauth A, Schemitsch E, Norris B, Nollin Z, Watson JT (2018). Critical-size bone defects: is there a consensus for diagnosis
and treatment?. J Orthop Trauma.

[31] Fayaz HC, Giannoudis PV, Vrahas MS, Smith RM, Moran C, Pape HC (2011). The role of stem cells in fracture healing and
nonunion. Int Orthop.

[B32] Cheung WH, Chin WC, Wei FY, Li G, Leung KS (2013). Applications of exogenous mesenchymal stem cells and low
intensity pulsed ultrasound enhance fracture healing in rat
model. Ultrasound Med Biol.

[B33] Li Y, Chen SK, Li L, Qin L, Wang XL, Lai YX (2015). Bone defect animal models for testing efficacy of bone substitute
biomaterials. J Orthop Transl.

[B34] Guimarães APFM, Butezloff MM, Zamarioli A, Issa JPM, Volpon JB (2017). Nandrolone decanoate appears to increase bone callus formation in
young adult rats after a complete femoral fracture. Acta Cir Bras.

[B35] Gholipour H, Meimandi-Parizi A, Oryan A, Bigham Sadegh A (2018). The effects of gelatin, fibrin-platelet glue and their
combination on healing of the experimental critical bone defect in a rat
model: radiological, histological, scanning ultrastructural and
biomechanical evaluation. Cell Tissue Bank.

[B36] Wu Y, Hanna EL, Holmes RE, Lin Z, Chiaramonti AM, Reeves RA (2018). External beam irradiation preferentially inhibits the
endochondral pathway of fracture healing: a rat model. Clin Orthop Relat Res.

[B37] Qu H, Fu H, Han Z, Sun Y (2019). Biomaterials for bone tissue engineering scaffolds: a
review. RSC Adv.

[B38] Caliogna L, Medetti M, Bina V, Brancato AM, Castelli A, Jannelli E (2021). Pulsed electromagnetic fields in bone healing: molecular pathways
and clinical applications. Int J Mol Sci.

[39] Hixon KR, Miller AN (2022). Animal models of impaired long bone healing and tissue
engineering- and cell-based *in vivo*
interventions. J Orthop Res.

[40] Oh SS, Narver HL (2024). Mouse and rat anesthesia and analgesia. Curr Protocols.

